# Neurosyphilis: The Great Imitator

**DOI:** 10.7759/cureus.32747

**Published:** 2022-12-20

**Authors:** Nina Jancar, Mariana Simões, Filipa Gonçalves, José Duro, Patricio Aguiar

**Affiliations:** 1 Internal Medicine, Hospital de Santa Maria, Lisbon, PRT

**Keywords:** autoimmune limbic encephalitis, acute confusional state, paraneoplastic encephalitis syndromes, neurosyphilis, cns lesions

## Abstract

Syphilis is a sexually transmitted disease caused by spirochete *Treponema pallidum*, with a growing incidence documented in recent years. Its clinical course is divided into three phases - primary, secondary, and tertiary syphilis - and virtually any organ can be affected, resulting in diverse clinical manifestations, making the diagnosis challenging. Neurosyphilis is a progressive, destructive disease of the central nervous system (CNS) that can develop at any stage of the infection, leading to meningeal involvement, meningovascular disease, or parenchymal syphilis (including tabes dorsalis and general paresis). Its clinical manifestations are heterogeneous and vary from focal neurologic signs to neuropsychiatric manifestations. The diagnosis is based mainly on the clinical picture and study of cerebrospinal fluid. Neuroimaging is helpful and sometimes essential, with magnetic resonance imaging being the most sensitive radiologic method, although there are no pathognomonic radiologic signs. Treatment of all forms of neurosyphilis is based on parenteral penicillin. We present a case of neurosyphilis in a patient presenting with a subacute confusional state and initial imaging findings suggestive of metastatic CNS lesions.

## Introduction

Neurosyphilis is a clinical manifestation of a sexually transmitted infection caused by the spirochete *Treponema pallidum*, presenting with variable and often unspecific symptoms [1,2]. Given the diversity of symptoms, it has been known as “the great imitator” throughout history. According to some studies, as much as half of the neurosyphilis cases are reported in patients with a concomitant human immunodeficiency virus (HIV) infection [3,4]. *Treponema* invades the central nervous system (CNS) in the first days after the infection [4] and can manifest itself in any phase of the disease [3,4]. It can be classified as symptomatic or asymptomatic; according to the time elapsed since the primary infection, it can be classified as early (one to two years after inoculation) or late [4,5]. Early infection is frequently characterized by aseptic meningitis or meningovascular meningitis. Late neurosyphilis manifests itself most frequently as parenchymal syphilis in the form of tabetic or paretic neurosyphilis, the latter characterized by heterogeneous psychiatric symptoms, which often leads to misdiagnosis of a primary psychiatric disease [3,4]. The diagnosis is challenging and is based on often heterogenous clinical manifestations and cerebrospinal fluid (CSF) and serum serologic tests - treponemal (FTA-ABS, for example) and nontreponemal (VDRL and RPR) tests with different levels of sensitivity and specificity [1,3-5]. Although there are no pathognomonic radiologic signs, neuroimaging is valuable and often essential for the diagnosis as well as the differential diagnosis of neurosyphilis [3,4]. We present a case of neurosyphilis in a patient presenting with a subacute confusional syndrome and initial imaging findings suggestive of metastatic CNS lesions.

This clinical case was presented in the form of a poster at the 27º Congresso Nacional de Medicina Interna (Villamoura, Portugal, October 2-5, 2021).

## Case presentation

We report a case of a 64-year-old woman, previously autonomous, with a medical history of essential arterial hypertension (medicated with an association of perindopril and amlodipine 10+10mg). She did not report any other relevant health conditions, previous history of sexually transmitted disease (including syphilis), previous interventions, or allergies. There was no history of risky sexual behavior or substance abuse (licit or illicit).

The patient’s symptoms began two months prior to the hospitalization when she started to present with periods of spatial and temporal disorientation, confusion, and loss of memory (mostly short-term). She was referred by her primary health care physician to perform a cranial computerized tomography (CT) scan, which showed digitiform hypodense lesions in the anterior regions of both temporal lobes, causing vasogenic edema, compatible with secondary CNS lesions, other digitiform foci of smaller dimensions in the fronto-opercular and fronto-polar paramedian regions in the right hemisphere and a meningioma of the right parietal lobe. She was referred to the neurosurgery outpatient clinic, based on the radiologic findings and clinical presentation.

About 15 days prior to the hospitalization, she developed new symptoms, namely generalized headache, episodic ear pain, worsening of disorientation and mental confusion, epigastric pain, and metrorrhagia, and sought medical attention at the emergency department. On the examination, disorientation, verborrhea, paraphasia, and visual and auditory hallucinations were denoted. The remaining observation was unremarkable, and she did not present any focal neurological signs.

**Figure 1:**
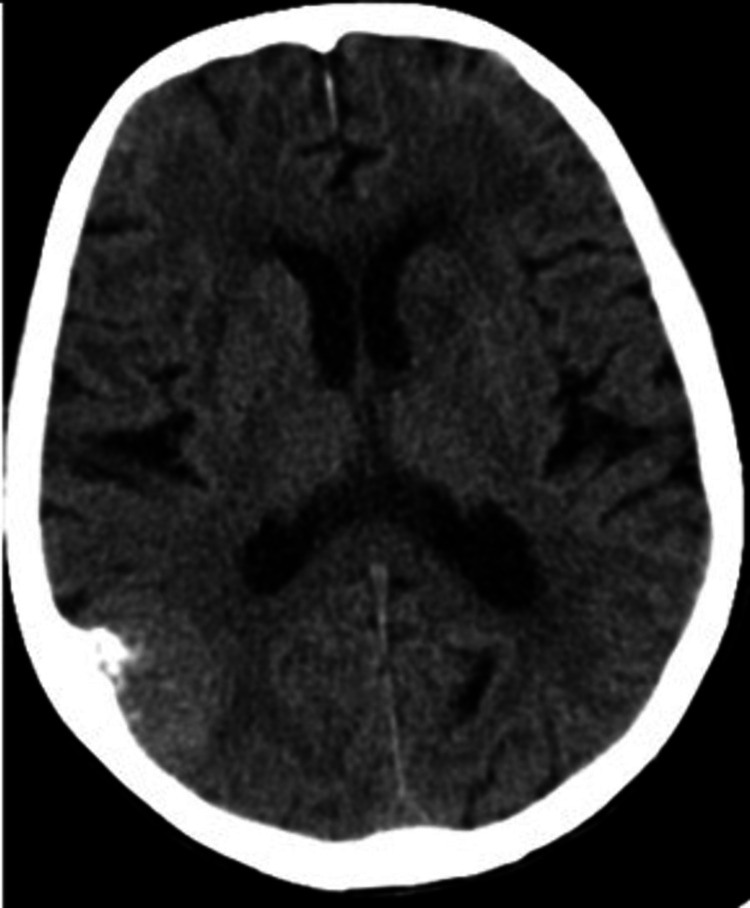
Cranial CT scan at admission, showing hypodense lesions in the anterior regions of both temporal lobes and a meningioma of the right parietal lobe. CT - computerized tomography

Further diagnostic tests revealed normocytic anemia (hemoglobin 11.9g/L and mean corpuscular volume (MCV) 88.7fL), without any other significant changes in blood chemistry (including urea, creatinine, liver tests, glucose, and serum protein); electrocardiogram with left anterior block and voltage criteria for left ventricular hypertrophy; cranial CT scan (Figure 1) showed no significant changes in comparison with the previous CT scan. She was admitted to the Internal Medicine department for further investigation of the cerebral lesions (suggestive of metastatic CNS lesions) and sub-acute confusional syndrome.

Given the radiologic findings suggestive of secondary CNS lesions, a pelvic exam, pelvic ultrasound (US), and cervical, thoracic, abdominal, and pelvic CT scan was conducted in order to identify the possible primitive neoplasm. The results were unremarkable.

Other etiologies of the subacute confusional syndrome were investigated, namely endocrine (thyroid function and thyroid gland US), nutritional (vitamin B12 and folate), infectious (Interferon-Gamma Release Assays [IGRA] test and blood serology for brucellosis, toxoplasmosis, borreliosis, cytomegalovirus [CMV], Epstein-Barr virus [EBV], herpes simplex type [HSV] 1 and 2, HIV and viral hepatitis B and C) and autoimmune (anti-nuclear antibodies [ANA], ANA profile, anti-phospholipid antibodies, anti-neutrophil cytoplasm antibodies, and rheumatoid factor), but the results were all unremarkable. An electroencephalogram was performed and ruled out the epileptic activity.

**Figure 2:**
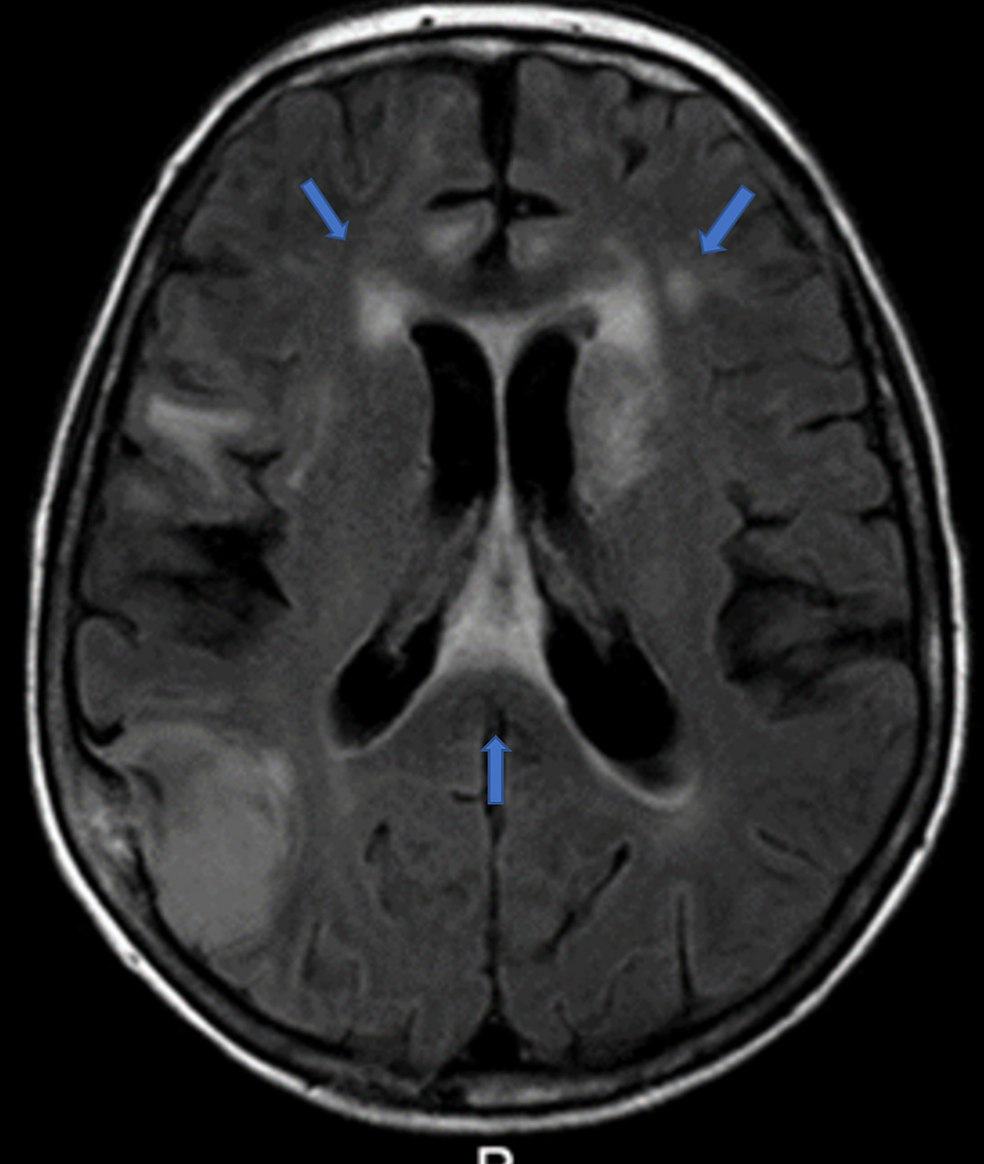
Cranial MRI scan (axial plane) showing hyperintense signal of subcortical white matter - bitemporal regions, mesial temporal lobe, corpus callosum, and internal capsule. Meningioma of the right parietal lobe. MRI - magnetic resonance imaging

Cranial magnetic resonance imaging (MRI) with gadolinium contrast was performed (Figure 2) and showed a hyperintense signal of subcortical white matter in the bitemporal regions, hippocampus, and mesial temporal lobes, as well as the involvement of corpus callosum, internal capsule, and diffuse involvement of the white matter in the frontal lobe. The radiologic findings suggested the following diagnostic hypothesis: CADASIL, autoimmune/limbic encephalitis, and multifocal progressive leukoencephalopathy.

In the course of the further etiologic investigation, a lumbar puncture (LP) was conducted, and it showed lymphocytic pleocytosis (20.8/mm^3^), as well as augmented protein levels (49.7mg/dL), high lgG level (128mg/dL) and RPR reactivity in the CSF. Bacteriologic and mycological exams of the CSF were negative (Table 1) and CMV, EBV, Cryptococcus, HSV1, and HSV2 were not detected. Brucellosis and Lyme disease serologies were negative in the CSF. The John Cunningham (JC) virus was not detected in CSF and there were no mutations detected in the NOTCH3 gene (Table 2), allowing us to exclude multifocal progressive leukoencephalopathy and CADASIL as a diagnostic hypothesis. 

**Table 1:** CSF biochemistry, cell count, nontreponemal tests, and CSF cultures CSF - cerebrospinal fluid

Lymphocytes	20.8/mm^3^
Proteins	49.7mg/dL
IgG	128mg/L
RPR	Reactive
VDRL	Reactive
Mycology test	Negative
Bacteriologic exam	Negative
Mycobacteria	Negative

**Table 2:** CSF serologies, JC virus PCR, and antineuronal antibodies. Autoimmune disease panel, NOTCH 3 gene sequencing, IGRA and syphilis serologies in peripheral blood. CSF - cerebrospinal fluid. IGRA - Interferon-γ release assay. PCR - polymerase chain reaction.

IGRA test	Not reactive
Brucella serology	Not reactive
Toxoplasmosis serology	Not reactive
Borrelia serology	Not reactive
CMV, EBV, HSV1, HSV2, HIV, Hepatitis C and B	Negative
JC virus	Negative
Autoimmune profile	Negative
NOTCH3 gene	Negative
Antineuronal antibodies	Negative
T. pallidum antibodies	Positive (19.45)
TPHA	Reactive(1/20480)
VDRL	Reactive
RPR	Reactive(128dils)

In light of the LP results, syphilis detection tests were conducted and showed positivity for RPR, TPHA, and VDRL assays, as well as *Treponema pallidum* antibodies in the patient’s serum, which confirmed the diagnosis of neurosyphilis. In addition, the patient’s serum was also tested for the presence of anti-neuronal antibodies.

The patient started treatment with penicillin G (24MU/day for ten days) and intravenous methylprednisolone pulses (1g/day, for five days) followed by oral prednisolone 1mg/Kg/day, as autoimmune encephalitis, which was one of the most important diagnostic hypotheses, was neither confirmed nor excluded at that time.

Anti-neuronal antibodies in the CSF were not detected. Although their clinical sensitivity is difficult to determine, they have a high negative predictive value [6]. Therefore, the hypothesis of autoimmune encephalitis was discarded, and corticotherapy was gradually tapered and discontinued.

The patient showed clinical improvement after treatment with penicillin, with no new episodes of confusion or altered mental status, and the blood titers of non-treponemal assays were lowered. In addition, cranial MRI was repeated three and six months after the treatment, and radiologic findings remained unchanged, as expected - according to some studies, the radiologic lesions remain stable or can even progress, despite the absence of symptoms [7].

## Discussion

Syphilis is a sexually transmitted disease caused by a gram-negative spirochete Treponema pallidum [1]. Although Portugal has the fifth lowest disease incidence in the European Union, a rise in the incidence has been registered in the last years [2].

Syphilis has a wide range of manifestations and is therefore known as the great imitator. Its clinical course can be divided into three phases: primary, secondary, and tertiary. Primary syphilis usually manifests after a 2-6-week incubation period as a painless skin/mucosal lesion (chancre) at the site of inoculation, associated with regional lymphadenopathy, and resolves spontaneously within 2-12 weeks. Secondary syphilis presents as generalized mucocutaneous lesions, initially discrete pink macules and papules involving the proximal extremities, palms, and soles, which can evolve into condyloma lata, and painless lymphadenopathy. It is often associated with constitutional symptoms (fever, malaise, weight loss, anorexia, and headache) and meningeal signs. Hepatic, kidney, gastrointestinal, and ophthalmologic involvement are fewer common manifestations of the disease. Like the primary form, it usually resolves spontaneously within one to six months. Patients in the latent phase have positive serologic tests for syphilis and are asymptomatic, but highly contagious. About one-third of patients with an untreated treponemal infection develop tertiary/late syphilis, with cardiovascular manifestations, gummatous disease, or neurologic manifestations [1].

Neurosyphilis is a destructive and progressive disease of the CNS. Although uncommon as early manifestation, it can develop at any stage of the disease, as soon as in the first weeks or months after the infection and can be followed by months to years of asymptomatic involvement, showing only CSF abnormalities, such as pleocytosis, increased protein concentrations or VDRL reactivity. When untreated, approximately 20%-30% of patients develop symptomatic neurosyphilis. The most common clinical entities of neurosyphilis are meningitis (common in the early stages of the disease), meningovascular syphilis, and parenchymal syphilis, which includes tabes dorsalis and general paresis. Clinical manifestations are heterogeneous and comprise headache, meningeal signs, involvement of the cranial nerves, strokes, gait ataxia, optic neuritis, and uveitis, as well as an array of neuropsychiatric manifestations, such as behavioral and personality disorders, dementia, and affective and cognitive disorders [1].

Prompt diagnosis of neurosyphilis can be challenging when based solely on the clinical picture, given the wide array of nonspecific symptoms. Although there are no pathognomonic radiological signs of neurosyphilis, there are some characteristic findings, which make radiologic methods a useful tool in the diagnostics of neurosyphilis, MRI being the most sensitive one [8].

Even though there are no pathognomonic radiologic signs, there are radiologic findings frequently associated with neurosyphilis [8]. Meningeo-vascular form, which is the most common one, does not have typical radiologic findings. It manifests itself as cerebral infarctions, most commonly of the cortico/subcortical area, thalamus/basal ganglia, and brain stem. Arteritis, usually of the medial cerebral artery and branches of the vertebral arteries, as well as leptomeningeal enhancement is also a frequent finding of the disease; we can distinguish two patterns of arteritis in neurosyphilis - Heubner arteritis, which is the more common form and affects the medium and large arteries, and the Nissl-Alzheimer form of arteritis, which affects the small arteries and arterioles. Cortical atrophy of frontal and temporoparietal lobes is the most common radiologic finding in tabes dorsalis and is associated with a poor prognosis, as well as diffuse white matter lesions with possible hyperintense T2 signal, that possibly traduces hyperpergliose and atrophy, and can be partially reversed with penicillin therapy. Bilateral hyperintense T2 signal in mesiotemporal lobes, however, was described only in a small number of patients and is a finding characteristic of herpetic encephalitis [8-10].

Given that our patient presented with sub-acute confusional syndrome and alteration of consciousness in conjunct with diffuse white matter lesions, most prominent in frontotemporal lobes and limbic structures on MRI, the most plausible diagnostic hypotheses were neurosyphilis and autoimmune encephalitis, upon exclusion of metabolic, neoplastic and most infectious causes of the acute confusional syndrome on the initial assessment.

Autoimmune encephalitis is an auto-antibody-mediated disease involving the CNS and can be classified into two large groups, based on the presence or absence of neoplastic disease. The most frequent cancer associated with autoimmune encephalitis is small-cell lung cancer, but can also occur in Hodgkin lymphoma, neuroblastoma, germ cell tumors, thymoma, breast cancer, and germ cell tumor of the testis; the other group is nonparaneoplastic autoimmune encephalitis. Paraneoplastic encephalitis is usually caused by antibodies against structures of the CNS (type 1 antibodies: e.g., anti-Hu, anti-Ma/Ta, anti-GAD) and the non-paraneoplastic form is more often associated with antibodies against cell-surface antigens (type 2: eg, anti-NDMA, anti-AMPAR, anti-gluR3) [11,12]. The latter group responds better to prompt immunomodulatory treatment and has a better prognosis [9]. Despite demonstrating a wide spectrum of clinical presentations ranging from the insidious onset of cognitive impairment to other forms of encephalopathy, both types present with characteristic radiologic findings primarily in the temporal lobe, especially in limbic structures. It is the second most common cause of encephalitis with temporal lobe abnormalities after herpes simplex virus and must be considered as a differential diagnosis of confusional syndrome with the described imaging findings [11-13].

## Conclusions

In conclusion, neurosyphilis can present with many different symptoms and various unspecific radiologic findings, which can be misleading in diagnosing neurosyphilis, as demonstrated in this case report. The CT image was highly suggestive of CNS metastasis. In contrast, the radiologic findings on MRI pointed towards the diagnostic hypothesis of CADASIL, limbic encephalitis, and multifocal progressive leukoencephalopathy, primarily because of the bilateral involvement of the mesiotemporal lobes, a radiologic finding not very common in neurosyphilis. Although it is not a very common finding, neurosyphilis should be suspected and ruled out in elderly or immunocompromised patients without a prior history of treponemal infection, presenting with a confusional syndrome and evidence of temporal lobe involvement on neuroimaging methods. Early diagnosis and treatment are beneficial.
